# Improved Adhesion of Bacterial Cellulose on Plasma-Treated Cotton Fabric for Development of All-Cellulose Biocomposites

**DOI:** 10.3390/molecules29215009

**Published:** 2024-10-23

**Authors:** Linda Ogrizek, Janja Lamovšek, Gregor Primc, Mirjam Leskovšek, Alenka Vesel, Miran Mozetič, Marija Gorjanc

**Affiliations:** 1 Faculty of Natural Sciences and Engineering, University of Ljubljana, Aškerčeva 12, 1000 Ljubljana, Slovenia; linda.ogrizek@gmail.com (L.O.); mirjam.leskovsek@ntf.uni-lj.si (M.L.); 2 Agricultural Institute of Slovenia, Hacquetova ulica 17, 1000 Ljubljana, Slovenia; janja.lamovsek@kis.si; 3 Jozef Stefan Institute, Jamova 39, 1000 Ljubljana, Slovenia; gregor.primc@ijs.si (G.P.); alenka.vesel@ijs.si (A.V.); miran.mozetic@guest.arnes.si (M.M.)

**Keywords:** all-cellulose biocomposite, bacterial cellulose, cotton, oxygen plasma, surface changes, adhesion

## Abstract

Cellulose produced by bacteria (BC) is considered a promising material for the textile industry, but the fragile and sensitive nature of BC membranes limits their broad applicability. Production of all-cellulose biocomposites, in which the BC is cultivated in situ on a cotton fabric, could solve this problem, but here a new issue arises, namely poor adhesion. To overcome this challenge, cotton fabric was modified with low-pressure oxygen plasma in either afterglow, E-mode, or H-mode. All-cellulose biocomposites were prepared in situ by placing the samples of cotton fabric in BC culture medium and incubating for 7 days to allow BC microfibril networks to form on the fabric. Modification of cotton fabric with oxygen plasma afterglow led to additional functionalization with polar groups, and modification with oxygen plasma in H-mode led also to etching and surface roughening of the cotton fibers, which improved the adhesion within the biocomposite. In addition, these biocomposites showed higher deformation capacities. Modification of the cotton fabric over a longer period in E-mode was found to be unsuitable, as this caused strong etching, which led to the defibrillation of cotton fibers and poor adhesion of BC. This study highlights the potential of low-pressure plasma treatment as an environmentally friendly method to improve the performance of cellulose-based biocomposites.

## 1. Introduction

A composite material consists of a combination of two or more components or phases with different physicochemical properties. It generally consists of two main phases: the matrix and the reinforcement [1]. Among composites, biocomposites are becoming increasingly popular because of their ecological advantages, as the reinforcing phase is of natural origin (e.g., cotton, flax, hemp, wood fibers) [2,3,4,5]. The properties of biocomposites depend on numerous parameters related to the complex interactions between the components. Of particular importance is the interfacial property that forms between the matrix and the reinforcement, as interfacial processes significantly influence the overall properties of composites [1]. The bonding at the interface is created by the adhesion of the components that are brought into close contact during the composite manufacturing process. In contemporary research, there is growing awareness of the need for sustainable solutions in the development of new materials that reduce negative impacts on the environment. In this area, the development of all-cellulose biocomposites, in which both phases (matrix and reinforcement) are of natural origin, is one of the most important research topics.

All-cellulose biocomposites are composed of two chemically identical components and were first described by Nishino et al. [6]. Since cellulose does not melt, the material must be dissolved to a regenerated form, which becomes the matrix phase of the composite. In most studies on all-cellulose composites, cellulose material is dissolved in NaOH, LiOH, urea, or deep eutectic solvents and then applied to solid cellulose [7,8,9,10,11]. During drying, the two phases combine chemically through the formation of hydrogen bonds. However, instead of dissolving a cellulose component, an all-cellulose composite can be prepared in situ by growing bacterial cellulose (BC) on the reinforcing phase (i.e., cellulose fabric or fibers) added at the beginning of the incubation, allowing a BC microfibril network structure to form around the reinforcing phase [12,13,14,15,16,17,18,19,20,21]. In these publications, better properties of the final composite material compared with the separate phase materials (i.e., increased hydrophilicity and tensile strength) were explained, but no significant analyses regarding adhesion were performed, although poor adhesion was observed during the experiments. In the production of composites, one of the phases is in the liquid state at some point. Therefore, the wettability of the reinforcing phase with the matrix has a significant influence on good adhesion and the formation of a bond between the reinforcing and matrix phases [22].

Gaseous plasma is considered an environmentally friendly medium for the surface modification of fibers, textiles, and other fibrous polymers because it does not require water or other solvents and because the amount of chemicals needed for surface treatment is minimal and often limited to nontoxic gases such as oxygen, air, and nitrogen [23]. Low-pressure inductively coupled radiofrequency plasma (RF ICP) is often used to modify textiles. This system does not require electrodes. Thus, contamination of the substrate with electrode material is avoided. An oscillating electric current flows through a coil and generates a homogeneous discharge [23]. The ICP discharge can have two modes of electron heating or power-coupled methods to sustain the plasma: the capacitively coupled method, or electrostatic electron heating mode (called E-mode), and the inductively coupled method, or electromagnetic (EM) electron heating mode (called H-mode) [24,25]. The mechanisms of interaction between oxygen plasma and textile samples are well documented in the literature [26,27,28]. It is reported that the interaction of atoms from the plasma is predominantly chemical, meaning that these atoms chemically bond with the surface of the substrate and form oxygen-rich functional groups. In addition to the substitution of atoms or simple radicals at the surface, positively charged ions cause significant bond breaking within the polymer chain, while VUV (vacuum ultraviolet) photons penetrate up to tens or even hundreds of nanometers into the cellulose and change the properties of relatively thick layers. VUV photons nonselectively break bonds and generate free radicals that facilitate structural modification of the substrate and lead to cross-linking within the polymer matrix. Oxygen plasma treatment enables advanced surface modification of textile materials by utilizing the unique properties of RF ICP, such as low-temperature processing, making it suitable for sensitive materials; high uniformity of treatment, enabling consistent processing over large areas; and the ability to selectively modify textile fibers.

It has previously been found that the expensive commercial growth medium for producing BC can be replaced by nutrients from white grape bagasse (waste from the wine industry) [29,30,31]. However, the BC membrane produced in grape bagasse medium [30] was rigid and brittle, limiting its usability in industries such as textiles and footwear and for applications such as filters, batteries, wound dressings, drug delivery carriers, packaging, and regenerative medicine [32]. It has been found that bacterial cellulose membranes can become brittle depending on biosynthesis and processing conditions [33]. To overcome the disadvantage of fragility of BC membranes, we prepared an all-cellulose biocomposite in which cotton fabric was added to the BC culture medium at the beginning of the BC synthesis process (in situ biocomposite preparation). Regarding the previously mentioned adhesion theory, we predicted that cotton, as a hydrophilic fiber, should already be suitable for the production of such biocomposites. However, we found that bacterial cellulose did not adhere well to the fabric despite the hydrophilic nature of cotton. Although the samples were carefully handled for the analysis, we found that the bacterial cellulose membrane grown on cotton was damaged and was easily detached from the fabric.

Other authors have suggested the use of sodium hydroxide or acetone or the addition of resin in the preparation of cellulose composites with bacterial cellulose [19,20,21]; however, this was not the subject of our research. Our aim was to develop a biocomposite as far as possible without hazardous chemicals. Therefore, the purpose of this study was to modify the surface of cotton fabric with gaseous plasma at low pressure, using oxygen as the working gas, to increase the adhesion between bacterial cellulose and cotton fabric in a biocomposite.

## 2. Results and Discussion

Despite the lack of studies on adhesion in all-cellulosic composites, it is known that adhesion is mainly governed by hydrogen bonding between two phases [22]. Theories on adhesion suggest that interfacial bond strength is enhanced by higher micro and macro surface effects (e.g., roughening of the reinforcing phase), leading to mechanical interlocking and frictional forces [34]. The phenomenon of bonding is, therefore, the sum of mechanical, physical, and chemical forces that overlap and influence each other. The purpose of our study was to increase adhesion in a biocomposite material made of cotton fabric and bacterial cellulose membrane by using oxygen plasma. Three types of plasma discharges were used to modify the cotton fabric, namely plasma flowing afterglow and E- and H-mode. The chemical changes on the surface of the cotton fabric after plasma treatment were analyzed by XPS, and the morphological changes were analyzed by SEM analysis. The surface composition was calculated from the XPS survey spectra. The results in Table 1 show that the plasma treatment increased the oxygen content and decreased the carbon content on the surface of the cotton fabric, which was reflected in the oxygen to carbon (O/C) ratio (Table 1). The untreated cotton sample (CO-UN) had about 65 at.% carbon and 35 at.% oxygen on its surface, and the corresponding O/C ratio was 0.5. This was well below the theoretical ratio of pure cellulose, which is about 0.8 [35]. Raw cotton fibers consist of 86% to 96% cellulose, depending on the degree of maturity and method of determination, while the other components of the fibers are noncellulosic substances (e.g., protein, wax, pectin and minerals) [36]. After scouring and bleaching (we used bleached and mercerized cotton fabric), most of the other components are removed, and the resulting cotton fibers have a purity of almost 99% of cellulose, resulting in an O/C ratio of about 0.5 to 0.6 [37]. The lower O/C ratio of the untreated cotton sample therefore indicated a lack of polar functional groups on the surface of the cotton samples and thus an inability to bind BC. When cotton was treated with oxygen plasma in H-mode (sample CO-H) and E-mode (sample CO-E), the surface of the cotton fabric was functionalized with oxygen-rich groups, as evidenced by a decrease in carbon content to about 55 at.% and an increase in oxygen content to about 44 at. %. Both samples had O/C ratios of 0.8, which was close to the theoretical value. In the treatment of the cotton sample with oxygen plasma afterglow (sample CO-A), the functionalization with oxygen-rich groups was even more pronounced, with an O/C ratio of 0.9. This large O/C ratio suggests the formation of highly polar, oxygen-rich functional groups, such as carboxylic groups, on the textile surface.

**Figure 1:**
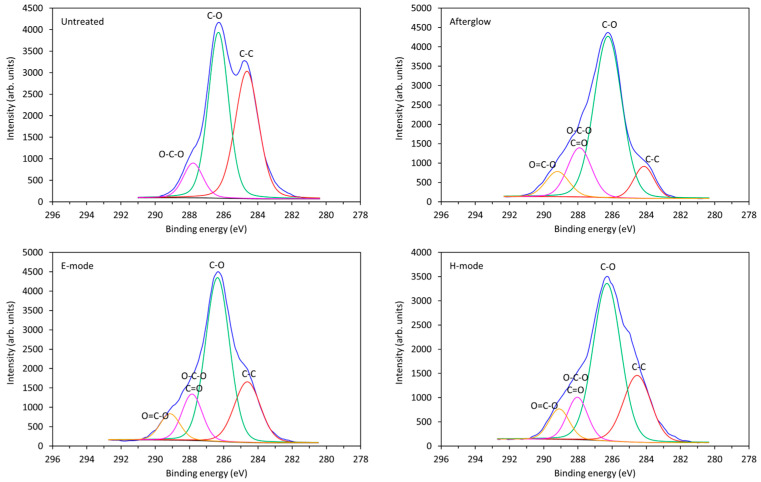
High-resolution XPS spectra of carbon (C 1s) with deconvoluted peaks for differently treated cotton fabric samples.

Additional information regarding the chemical changes on the surface of the cotton fabric after plasma treatment and the chemical bonding of the elements on the surface was obtained from high-resolution XPS spectra (Figure 1). The peaks in Figure 1 consist of several subpeaks that belong to the specific bindings of C atoms on the surface: C–C bond, C–O bond, O–C–O or C=O bond, and O=C–O bond. The peaks were deconvoluted, and the approximate relative concentrations of these bonds are listed in Table 2.

The concentrations of C-O and C-C bonds on the CO-UN sample were similar, although the concentration of C-O bonds was slightly higher. The CO-UN sample also contained a small amount of O-C-O/C=O bonds. Oxygen plasma treatment increased the percentage of O-C-O/C=O bonds on the surfaces of all of the treated samples. The highest increase was observed for sample CO-A (17.1%), and the lowest, for sample CO-H (11.3%). Plasma treatment also led to the formation of new O=C-O bonds, with the largest proportion on the surface of sample CO-A (8.5%), followed by sample CO-H (8.1%) and sample CO-E (7.5%), but the differences were within the limits of experimental error. Accordingly, the ratio of C-C bonds was strongly reduced in all plasma-treated samples. The observed increase in the O/C ratio was expected, since oxygen plasma treatment leads to oxidation of the surface and promotes the formation of oxygen-rich functional groups there, although the functionalization rate depends on the plasma system and the discharge parameters [38]. In our case, the strongest functionalization with oxygen-rich groups was achieved via afterglow discharge. This was the least intense plasma discharge, generated at 30 cm from the RF coil and characterized by a low electron density but a high density of neutral reactive species, especially O atoms.

**Figure 2:**
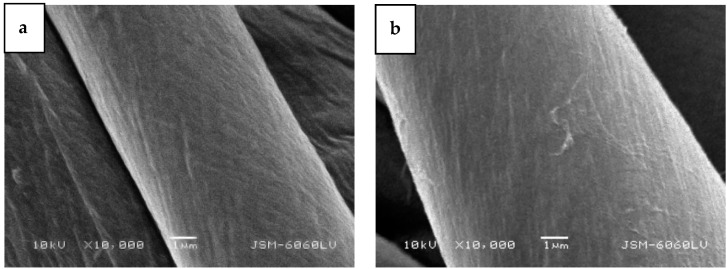

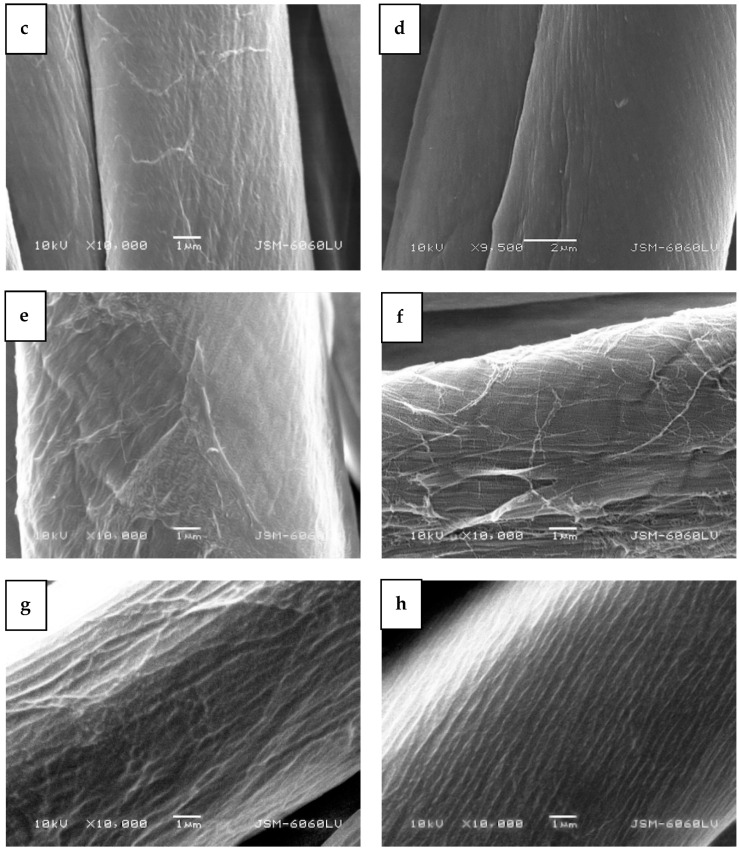
SEM images of bleached, mercerized cotton fabric samples: (**a**,**b**) untreated, (**c**,**d**) treated with plasma afterglow, (**e**,**f**) treated with plasma in E-mode, (**g**,**h**) treated with plasma in H-mode.

**Figure 3:**
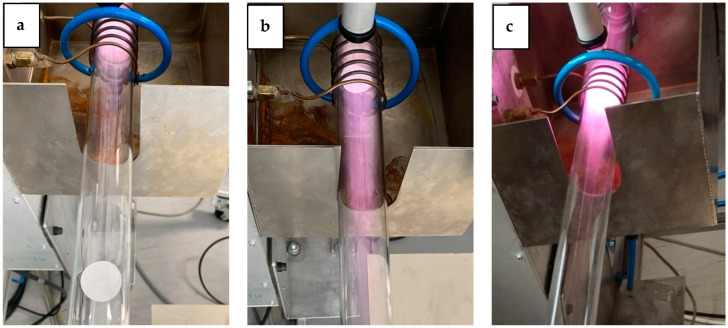
Light emission of a plasma discharge: (**a**) afterglow, (**b**) E-mode, (**c**) H-mode.

The SEM images in Figure 2 show surface morphological changes on cotton fibers after plasma treatment. As already mentioned, the topography of the reinforcement phase in the biocomposite is important for good adhesion. The SEM image of the untreated cotton fabric (sample CO-UN) shows the characteristic grooved surface morphology of mercerized cotton fibers with visible macrofibrils, which are predominantly oriented in the fiber axis direction and smooth and distinct because of the amorphous layer covering the fiber. The surface morphology of the plasma-treated fibers varied depending on the specific conditions of plasma treatment. The afterglow plasma was weak, so it did not cause any significant changes in topography (Figure 2c,d), rather primarily facilitating surface functionalization (Table 1 and Table 2). Treatment of cotton fabric with oxygen plasma in E-mode led to significant surface etching of the fibers, with the top layer peeling off and individual fibrils detaching, as can be seen in Figure 2e,f. The etching effect was also observed in samples treated with oxygen plasma in H-mode (Figure 2g,h), but here the fibers still had transverse connections between individual macrofibrils in the primary cell wall. The outlines of the macrofibrils were sharp and distinct. In an oxygen plasma environment, chemical functionalization and etching can occur, because the density of charged particles is low compared with the density of neutral oxygen atoms, which are highly reactive and interact with the surfaces of treated substrates at low temperatures [23]. Interaction with oxygen atoms leads to functionalization with oxygen-rich groups, while prolonged exposure leads to gradual surface etching. The significant etching induced by oxygen plasma in E-mode (Figure 2e,f) is likely to deteriorate the mechanical properties of the fabric. According to other studies, strong plasma etching of cotton fabric can reduce tensile strength, while mild etching can improve it [39,40]. The intensity of the plasma discharge can also be seen from images taken during the experiment (Figure 3). The weakest light emission due to plasma activity can be seen in Figure 3a, which shows the cotton fabric sample treated with plasma afterglow. The most intense light emission can be seen in Figure 3c, where the sample was treated with plasma in H-mode. Plasma in H-mode is intense and concentrated within the coil volume, with a high density of charged particles and an oxygen atom density three times higher than that of plasma in E-mode [41], so a short treatment (1 s) resulted in uniform surface etching of the fibers without visible damage (Figure 2g,h) and functionalization with oxygen-rich groups (Table 1 and Table 2). The etching in E-mode was about 100 times more intensive than that in afterglow treatment [42]. Etching in afterglow treatment is very slow [43], so the surface of the substrate remains saturated with polar groups despite the high flux density of oxygen atoms [44]. The chosen plasma parameters are explained in more detail below.

We chose plasma treatment parameters to take advantage of the different reactants that dominate the oxygen plasma in each of the three modes. The power density in H-mode (i.e., the absorbed power in a volume of glowing plasma) is so large that the oxygen plasma is a significant source of charged particles, metastables, and radiation. Figure 3c shows extensive radiation in the visible range, but radiation in the vacuum ultraviolet range (photon energy above 6 eV) predominates in such strong discharges. The neutralization of charged particles (positive ions and electrons), the relaxation of metastable (atomic and molecular) particles, and the absorption of low-wavelength radiation are all strongly exothermic surface reactions. Therefore, any material placed in a dense plasma in H-mode will heat up considerably without chemical surface reactions such as oxidation. Therefore, the advantage of treatment in H-mode is extensive bond breaking in the surface of the cellulose due to absorption of the VUV radiation and strong chemical reactions of highly excited atomic metastables. On the other hand, the extensive exothermic reactions on the surface lead to an increase in sample temperature, so we chose a treatment of 1 s as a compromise between surface reactions and permissible heating. The plasma in E-mode is shown in Figure 3b. The sample is clearly visible, so the plasma was not a significant source of visible radiation. The low radiation was a consequence of the relatively low electron density due to the poor coupling between the RF coil and the plasma in the discharge tube. The power of 110 W is the value displayed on the RF generator (a standard method for determining plasma power). However, because of the low electron density, and therefore poor conductivity, of the E-mode plasma, most of the power supplied by the generator was not absorbed by the plasma but reflected or radiated. The results of the inadequate power absorption were a low ionization fraction, a low concentration of highly excited metastables, and low radiation. However, the dissociation fraction was high. The loss of atoms in the gas phase was negligible at the selected pressure, and the production of atoms by electron impact dissociation of neutral oxygen molecules was high. This was due to the relatively low dissociation energy of 5.2 eV and the presence of metastable molecules with long lifetimes in the oxygen plasma, such that the dissociation was a two-step reaction that could be carried out with electrons of relatively low energy. E-mode plasma causes significant etching at low temperatures because of the synergy between the radiation, metastable molecules, and ground-state oxygen atoms. The treatment time can be extended without the risk of overheating, so we chose 20 s. The third mode was afterglow. Figure 3a shows no visible radiation at the sample position, which means that only long-lived plasma species were present. Therefore, the samples were treated with neutral atoms in the ground state and molecular metastables. The interaction did not benefit from synergistic effects with higher-energy plasma species, so the etching was slow, and the temperature remained constant even with long treatment times. The lack of exothermic reactions at the surface provided excellent conditions for surface functionalization at room temperature, so we used this mode to ensure saturation of the cotton samples with polar functional groups. The treatment time of 60 s was chosen to ensure optimal functionalization of the surface even in gaps.

The effects of chemical (functionalization) and physical (etching) modifications of cotton fabric by plasma treatment on the properties of all-cellulose biocomposites between cotton and bacterial cellulose membrane were investigated by SEM analysis, DMA analysis, and adhesion testing.

**Figure 4:**
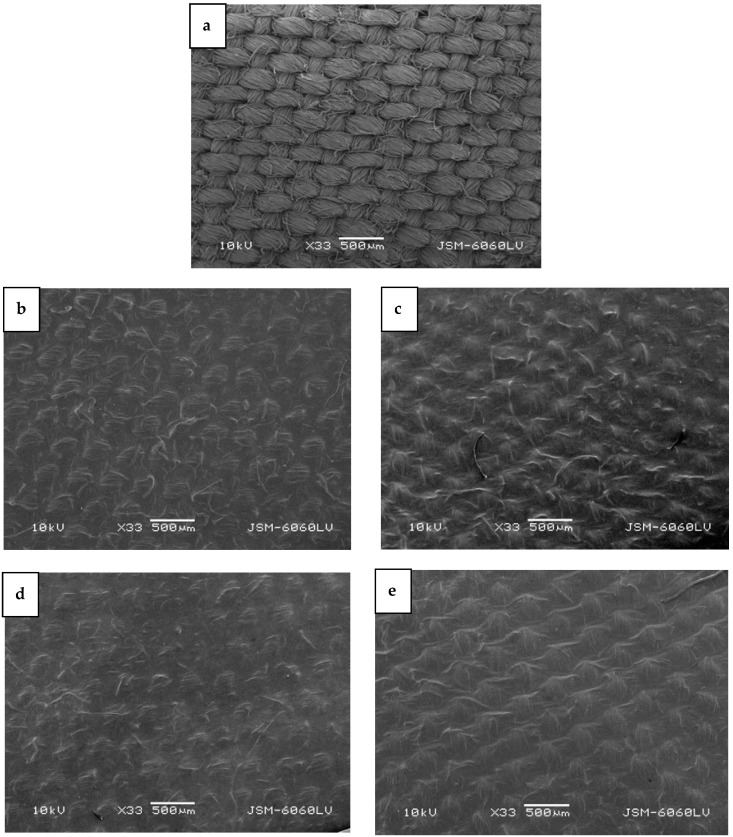
SEM images of (**a**) bleached, mercerized cotton fabric; (**b**) to (**e**) all-cellulose biocomposites prepared with cotton fabric that was (**b**) untreated, (**c**) treated with plasma afterglow, (**d**) treated with plasma in E-mode, and (**e**) treated with plasma in H-mode.

In cases where cotton fabric was incorporated into the bacterial cellulose medium to form a biocomposite (Figure 4b–e), the typical surface characteristic appearance of plain woven fabric (Figure 4a) was reduced. However, the biocomposite sample in which cotton fabric treated with plasma in H-mode was used (Figure 4e) was visually different from the other biocomposites (Figure 4b–d), as the BC membrane was formed more uniformly on the fabric, giving the biocomposite a more homogeneous appearance.

The all-cellulose biocomposites and the BC membrane were investigated with regard to their elastic (storage) moduli (E′), loss moduli (E″), and damping (tan δ) at room temperature (Table 3) to gain the mechanical stiffness and energy absorption when subjecting the samples to isothermal oscillating mechanical load. All the samples showed much higher storage moduli than loss moduli; therefore, the BC membrane and all-cellulose biocomposites could be regarded as mainly elastic. The BC membrane was the stiffest (highest E′) but also had the highest vulnerability to mechanical deformation (highest E″), indicating the highest structural friction of all samples. By combining cotton fabric and BC into an all-cellulose biocomposite, we obtained a material with greater deformability on the micron level of the structure, as storage moduli decreased. Damping (tan δ), which provides information on overall flexibility and represents the ratio of the viscous to the elastic response of the structure, showed overall very low values in the BC membrane and all-cellulose biocomposites (in the range of 0.058 to 0.090). The energy dissipation potential was therefore low for all samples, meaning that viscous mechanisms had a small influence on the final properties of the material. The composite prepared with H-mode plasma-treated cotton (sample CO-H-BC) showed the highest tan δ (0.136), indicating that at room temperature, the material was the most flexible (the least stiff) and dissipated the highest amount of energy of all the samples.

**Figure 5:**
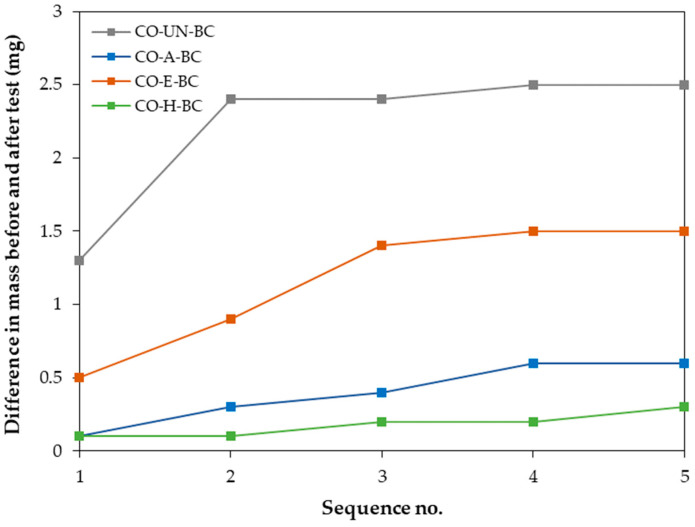
Difference between the initial mass of the biocomposite and the mass of the sample after completion of the adhesion test.

For an all-cellulose biocomposite to be functional, it must have strong adhesion between the matrix (BC membrane) and the reinforcing phase (cotton fabric), as well as improved elasticity under load. In reviewing the literature on testing the adhesion of thin coatings to various substrates, it was found that the most commonly used method is the tape method described in ASTM D3359, “Standard Test Methods for Rating Adhesion by Tape Test”. In this method, a piece of adhesive tape is applied to the surface of a damaged sample and then quickly peeled off to visually rate the adhesion on a scale of zero to five [45,46,47,48,49]. As this method provides qualitative rather than quantitative results, some researchers have adapted the test to obtain more specific adhesion ratings [11,50,51]. According to the ASTM D3359 method, the best adhesion (5A) was rated for the biocomposite sample in which cotton treated with H-mode oxygen plasma was used (sample CO-H-BC), followed by the biocomposite sample in which cotton treated with plasma afterglow was used (sample CO-A-BC, adhesion rating 4A) (Table 4). Low adhesion ratings (1A) were found for the samples in which untreated and E-mode plasma-treated cotton fabric were used. In addition, we performed a quantitative assessment. To quantify the adhesion within a biocomposite, the weight of the sample was measured both before and after up to five tape-test sequences (although the standard specifies only an assessment after a single sequence). The results of the quantitative adhesive tape test (Figure 5) clearly showed poor adhesion for the CO-UN-BC and CO-E-BC samples and good adhesion for the CO-A-BC and CO-H-BC samples. The difference in the mass of the BC membrane removed from the cotton fabric within the biocomposite was greatest for the sample where untreated cotton fabric was used, wherein most of the BC membrane covering the cotton fabric was removed after the tape was first pulled (Figure 5). For the CO-E-BC biocomposite, where a cotton fabric treated with plasma in E-mode was used as reinforcement, the removal of the BC membrane was greater after the second test sequence than after the first. Very gradual removal, with very little difference in mass before and after the tape test, was observed in the biocomposites with the best adhesion ratings (samples CO-A-BC and CO-H-BC).

As mentioned in the introduction, the wettability (hydrophilicity) of the reinforcement is the first step for good adhesion and bond formation. Since bleached and mercerized cotton fabric is inherently very hydrophilic, the contact angle cannot be measured [52]. From this point of view, the condition for adhesion should already be fulfilled for biocomposites made of bacterial cellulose and cotton fabric. However, as can be seen from our results, this was not the case. To achieve good adhesion, a higher concentration of oxygen-rich functional groups on the cotton fabric is required. Even more important, however, is increased roughness of the fibers; because of the shrinkage of the BC hydrogel drying on the cotton fabric during the production of the biocomposite, the increased frictional force between the hydrogel and the fabric contributes to better mechanical bonding [53]. Although we found higher oxygen functionalization and etching effects on the cotton fabric treated with plasma in E-mode, the adhesion within the biocomposite (sample CO-E-BC) was rather poor. The reason for this was excessive etching of the fibers, which led to delamination of the fibrils, as described earlier. In this sample, it is likely that in addition to the BC membrane, the delaminated fibrils of the cotton fibers were also removed, resulting in a poor adhesion rating as determined by the tape test.

**Figure 6:**
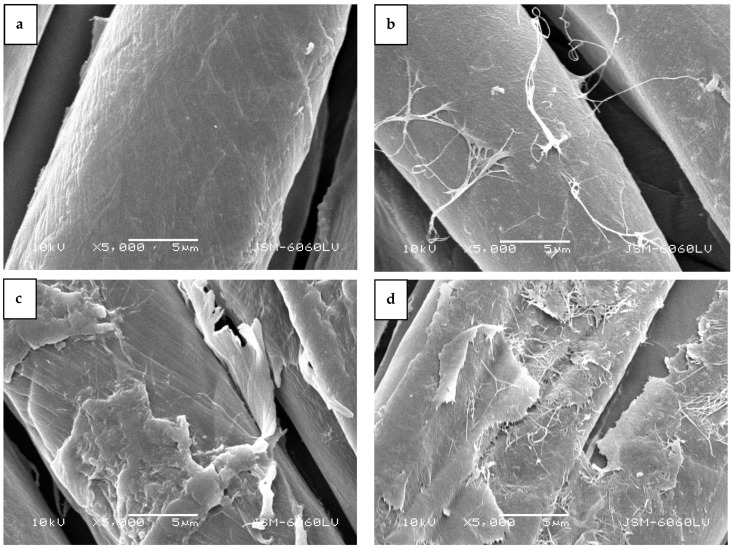
SEM images of samples after tape test: (**a**) CO-UN-BC, (**b**) CO-A-BC, (**c**) CO-E-BC, and (**d**) CO-H-BC.

The SEM images in Figure 6 depict the surface morphology of cotton fibers after the adhesive tape test. In the untreated cotton sample (Figure 6a), the BC membrane was completely removed, revealing the smooth and typical morphology of bleached and mercerized cotton fibers. Similarly, the afterglow-treated sample showed the typical cotton fiber morphology, with some remnants of bacterial cellulose fibrils protruding from the fiber surface (Figure 6b). In the H-mode-treated sample, more remnants of the BC membrane were visible (Figure 6d). Here, the cotton fiber morphology was less distinct, as the remaining BC membrane covered the fibers. Several bacterial cellulose fibrils clearly protruded from the membrane, obscuring the characteristic morphology of the cotton fibers.

In the E-mode-treated sample, where poor adhesion was associated with the removal of the delaminated cotton fibrils, SEM images in Figure 6c reveal that not only were the surface fibrils removed along with the BC membrane, but the primary wall of the cotton fiber was torn away, exposing the secondary wall of cotton fiber. The mesh structure of the primary wall was no longer continuous, and the spiral layer inside the fiber at the protoplasmic boundary, with helix angles of approximately 35°, was clearly visible [54].

## 3. Materials and Methods

### 3.1. Materials

The bacterial strain *Komagataeibacter xylinus* DSM 6513 (Leibniz Institute DSMZ, Brunswick, Germany), white grape bagasse extract (Agricultural Institute of Slovenia, Ljubljana, Slovenia), and bleached and mercerized 100% cotton fabric ((CO), plain weave, weight 110 g/m^2^, number of warp threads: 60 threads/cm, number of weft threads: 32 threads/cm (Tekstina, Ajdovščina, Slovenia)) were used for the study.

### 3.2. Plasma Treatment of Cotton Fabric

**Figure 7:**
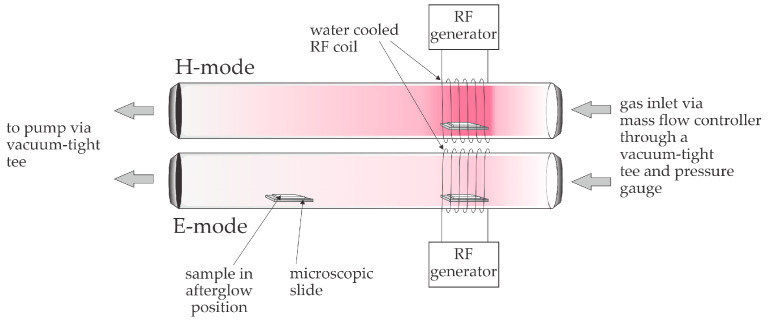
Schematic representation of the plasma system.

The cotton fabric used as reinforcement for the cellulose biocomposites was treated in a low-pressure radiofrequency inductively coupled plasma system (Figure 7). For all treatments, oxygen gas was used at a pressure of 38.2 Pa. The cotton fabric was cut into circular samples of 2.5 cm diameter. The power and duration of the plasma treatment were varied, as shown in Table 5.

### 3.3. In Situ Production of All-Cellulose Biocomposites

All-cellulose biocomposites were prepared in situ by placing cotton fabric in a bacterial cellulose growth medium (white grape bagasse) at the beginning of the BC synthesis process, as described in [30]. The experimental setup was carried out under aseptic conditions, and six biocomposite samples were prepared within one modification of the cotton fabric (untreated (CO-UN-BC) and treated with plasma in afterglow (CO-E-BC), E-mode (CO-E-BC), and H-mode (CO-H-BC)). After the 7-day cultivation period, the prepared biocomposites were rinsed in distilled water and immersed in 0.1 M NaOH for 120 min at 80 °C with gentle shaking to remove any attached bacterial cells and impurities. The biocomposites were rinsed in distilled water to remove NaOH and dried for 34 h on a hydrophobic plate at 50 °C.

### 3.4. Scanning Electron Microscopy (SEM)

The morphological characteristics of untreated and plasma-treated cotton fabrics and all-cellulose biocomposites were examined using a scanning electron microscope (SEM) (JSM-6060LV, JEOL, Tokyo, Japan). The samples were attached to copper rods with double-sided adhesive tape and sputter-coated with gold. The SEM images were taken at different magnifications, a beam voltage of 10 kV, a working distance of 27 mm, and a beam spot size of 30 nm.

### 3.5. X-Ray Photoelectron Spectroscopy (XPS)

The chemical composition of the surface of the untreated and plasma-treated cotton samples was characterized by X-ray photoelectron spectroscopy (XPS instrument TFA XPS, Physical Electronics, Feldkirchen near Munich, Germany). The samples were irradiated with monochromatic Al Kα1,2 radiation at 1486.6 eV over 400 µm^2^. The photoelectrons were detected using a hemispherical analyzer positioned at an angle of 45° relative to the sample surface. The XPS spectra were obtained at a transition energy of 187 eV with an energy step of 0.4 eV. The high-resolution XPS spectra of C1s carbon were measured at a transition energy of 29.35 eV with an energy step of 0.125 eV. An additional electron source was used to neutralize the surface charge. The measured spectra were analyzed using MultiPak v8.1c software (Physical Electronics, Feldkirchen near Munich, Germany). The spectra of C1s were fitted with a Gauss–Lorentz function.

### 3.6. Dynamical Mechanical Analysis (DMA)

Dynamical mechanical analysis of the BC membrane and the untreated and plasma-treated all-cellulose biocomposites was performed using a dynamic mechanical apparatus, DMA Q800 (TA Instruments, New Castle, DE, USA). The tension isothermal mode of deformation was performed at room temperature (24 °C), isothermal time 5 min, with an amplitude of 5 μm and at a frequency of oscillation of 10 Hz. The samples were cut in rectangular shapes with the following dimensions: approx. 6 mm (W) × approx. 20 mm (L), with a measuring clamping length of approx. 10 mm. The storage (E′) and loss (E″) moduli and damping (tan δ) were determined isothermally.

### 3.7. Adhesion Assessment of Biocomposites Using the Tape Test Method

The adhesion between the BC membrane and the cotton fabric within the all-cellulose biocomposites was tested according to ASTM D3359 (“Standard Test Methods for Rating Adhesion by Tape Test”) [45] with slight modification. Each sample was folded in half three times to cause damage to the BC membrane, covered with adhesive tape (Scotch 3M, 19 mm wide and 50 mm long), and smoothed. After 90 s, the tape was removed by grasping the free end and quickly peeling it off over itself at an angle of 180°. The adhesion rate was evaluated according to the ASTM scale from 0 to 5. In addition, the samples were weighed before and after the test to quantify the adhesion rating. Tests were performed in triplicate.

## 4. Conclusions

Oxygen plasma treatment was used to modify cotton fabric. The plasma-treated samples were immersed into growing broth for in situ preparation of all-cellulose biocomposites with bacterial cellulose. Chemical and morphological surface changes of the cotton fibers after plasma treatment were investigated to explain the importance of oxygen-rich functionalization and etching of the fiber surface, which led to the improvement of adhesion between the fabric and bacterial cellulose within the biocomposites. Three plasma operational modes (afterglow, E-mode, and H-mode) were used in the modification of the cotton, each of which contributed to different properties of the resulting biocomposites. The results showed that the plasma treatment, especially in H-mode and afterglow, significantly improved the adhesion between the BC membrane and the cotton fabric within the biocomposite. This was primarily due to the chemical functionalization of the cotton surface with oxygen-rich groups and the controlled etching that increased the surface roughness and thus improved the mechanical interlocking between bacterial cellulose fibrils and cotton fibers. Although the E-mode treatment resulted in surface functionalization and an etching effect, adhesion within the biocomposite was poor because of the defibrillation of the fibers. In addition, DMA results showed that the BC membrane sample still showed the highest stiffness when compared with the all-cellulose biocomposites (untreated or plasma-treated), but the viscous components of the structures showed only a small influence on the final elastic properties of the untreated and plasma-treated all-cellulose biocomposites at room temperature. Overall, the study provides valuable insight into optimizing plasma treatment parameters to improve the performance of cotton and bacterial cellulose biocomposites. Future work could investigate the long-term durability of these biocomposites and their behavior under different environmental conditions.

## Figures and Tables

**Table 1:** Chemical composition of the surface of untreated (CO-UN) and plasma-treated cotton fabric samples in the afterglow (CO-A), E-mode (CO-E), and H-mode (CO-H) (in at. %).

Sample	C	O	O/C
CO-UN	65.1	34.9	0.5
CO-A	51.9	48.1	0.9
CO-E	55.4	44.6	0.8
CO-H	55.9	44.1	0.8

The O/C ratio of pure cellulose is about 0.8 [35,36].

**Table 2:** Bonding of carbon atoms (concentration in %) obtained by fitting the high-resolution XPS spectra curves of untreated (CO-UN) and plasma-treated cotton fabric samples in the afterglow (CO-A), E-mode (CO-E), and H-mode (CO-H).

Samples	C–C	C–O	O–C–O/C=O	O=C–O
CO-UN	43.0	47.4	9.7	-
CO-A	9.4	64.9	17.1	8.5
CO-E	22.7	56.3	13.5	7.5
CO-H	24.0	56.6	11.3	8.1

**Table 3:** The elastic modulus (E′), loss modulus (E″), and damping (tan δ) of bacterial cellulose membrane (BC membrane) and all-cellulose biocomposites prepared in situ with bacterial cellulose and untreated (CO-UN-BC) and plasma-treated cotton fabric (CO-A-BC: afterglow, CO-E-BC: E-mode, CO-H-BC: H-mode).

Sample	E′ (GPa)	E″ (GPa)	tan δ
BC membrane	0.216	0.015	0.068
CO-UN-BC	0.058	0.006	0.058
CO-A-BC	0.070	0.006	0.079
CO-E-BC	0.086	0.008	0.090
CO-H-BC	0.035	0.005	0.136

**Table 4:** Evaluation of adhesion within all-cellulose biocomposites between the BC membrane and untreated (CO-UN-BC) and plasma-treated (CO-A-BC: afterglow, CO-E-BC: E-mode, CO-H-BC: H-mode) cotton fabric by the tape method.

Sample	Adhesion Rating
CO-UN-BC	1A
CO-A-BC	4A
CO-E-BC	1A
CO-H-BC	5A

**Table 5:** Plasma parameters according to treatments.

Mode of Operation	Time (s)	Power (W)	Distance of CO Sample from Coil (cm)
Afterglow	60	90	30
E-mode	20	110	0
H-mode	1	350	0

## Data Availability

Data are contained within the article.
